# Growth performance, serum biochemical profile, jejunal morphology, and the expression of nutrients transporter genes in deoxynivalenol (DON)- challenged growing pigs

**DOI:** 10.1186/s12917-015-0449-y

**Published:** 2015-07-03

**Authors:** Li Wu, Peng Liao, Liuqin He, Wenkai Ren, Jie Yin, Jielin Duan, Tiejun Li

**Affiliations:** Institute of Subtropical Agriculture, Chinese Academy of Sciences, 644# Yuandaer Road, Changsha, 410125 China

**Keywords:** Deoxynivalenol (DON), Amino acid transporter, Growing pigs, Nutrition

## Abstract

**Background:**

*Fusarium* infection with concurrent production of deoxynivalenol (DON) causes an increasing safety concern with feed worldwide. This study was conducted to determine the effects of varying levels of DON in diets on growth performance, serum biochemical profile, jejunal morphology, and the differential expression of nutrients transporter genes in growing pigs.

**Results:**

A total of twenty-four 60-day-old healthy growing pigs (initial body weight = 16.3 ± 1.5 kg SE) were individually housed and randomly assigned to receive one of four diets containing 0, 3, 6 or 12 mg DON/kg feed for 21 days. Differences were observed between control and the 12 mg/kg DON treatment group with regards to average daily gain (ADG), although the value for average daily feed intake (ADFI) in the 3 mg/kg DON treatment group was slightly higher than that in control (*P*<0.01). The relative liver weight in the 12 mg/kg DON treatment group was significantly greater than that in the control (*P*<0.01), but there were no significant differences in other organs. With regard to serum biochemistry, the values of blood urea nitrogen (BUN), alkaline phosphatase (ALP), alanine aminotransferase (ALT) and aspartate amino transferase (AST) in the 3 treatment groups were higher than those in the control, and the serum concentrations of L-valine, glycine, L-serine, and L-glutamine were significantly reduced in the 3 treatment groups, especially in the 12 mg/kg DON group (*P*<0.01). Serum total superoxide dismutase (T-SOD), glutathione peroxidase (GSH-Px) were markedly decreased after exposure to DON contaminated feeds (*P*<0.01). The villi height was markedly decreased and the lymphocyte cell numbers markedly increased in the 3 DON contaminated feeds (*P*<0.01). The mRNA expression levels of excitatory amino acid transporter-3 (EAAC-3), sodium-glucose transporter-1 (SGLT-1), dipeptide transporter-1 (PepT-1), cationic amino acid transporter-1 (CAT-1) and y^+^L-type amino acid transporter-1 (LAT-1) in control were slightly or markedly higher than those in the 3 DON treatment groups.

**Conclusions:**

These results showed that feeds containing DON cause a wide range of effects in a dose-dependent manner. Such effects includes weight loss, live injury and oxidation stress, and malabsorption of nutrients as a result of selective regulation of nutrient transporter genes such as EAAC-3, SGLT-1, PepT-1, CAT-1 and LAT-1.

## Background

The trichothecene deoxynivalenol (DON) is a secondary metabolite mainly produced by the plant pathogens *Fusarium graminearum* and *Fusarium culmorum*, to which human and livestock can be exposed via food and feed [1]. *Fusarium* infection of wheat, barley and corn with concurrent production of DON and other trichothecene mycotoxins is an increasing food safety concern worldwide [1, 2]. Many published papers show the toxic effects of DON on animals mainly impairing immune system, health status of the gastrointestinal tract and the brain [1, 3–6]. Some reports suggested that ingestion of these DON may induce feed refusal, organ damage, increased disease incidence, and malabsorption of nutrients [1, 3, 7–13]. Some papers showed in the in vitro studies that DON interfere with differentiation of different intestinal cell line models [10–14]. In vivo, much attention has been given to the determination of glucose absorption after DON exposure but only limited studies have assessed expression of nutrient transporter genes when feeding functional nutrients to alleviate poisoning triggered by a single dose of dietary DON exposure [9, 15–22]. DON is effectively absorbed in the upper gastro-intestinal tract (GIT), i.e. stomach, duodenum and proximal jejunum [23]. It is, therefore, hypothesized that DON will impair absorption of nutrients including amino acid, di/tripeptides, and glucose by reducing expression of genes for transporters of these nutrients especially in the upper GIT. Duration and amount of DON exposure seem to be crucial factors for toxic effect on nutrient digestibility and absorbability as previously shown in swine and cell line models [23, 24]. However, there has been no systematic investigation to date of the DON-triggered effects in growth performance, serum parameters, jejunal morphology, and in the expression of nutrient transporter genes.

Therefore, the objective of the present study was to investigate the effects of various levels (0 to 12 mg/kg) of dietary DON challenge on growth performance, serum biochemical and amino acid profile, jejunal morphology, and the differential expression of genes for nutrient transporters in growing pigs.

## Results

### Growth performance

The cumulative performance results of growing pigs are showed Table 1. There was no significant difference between control, 3 mg/kg DON group, and 6 mg/kg DON groups with regard to average daily gain (ADG), but this value in 12 mg/kg DON groups was significantly lower than those in the other groups (*P* < 0.05). The average daily feed intake (ADFI) was no significant difference between control and 3 mg/kg DON group, but this value in 6 mg/kg DON group and 12 mg/kg DON group were significantly lower than control and 3 mg/kg DON group (*P* < 0.05). The 6 mg/kg DON group showed the lowest feed/gain ratio (F/G).

**Table 1:** Growth performance of growing pigs fed with diets deoxynivalenol (DON) -contaminated corn from 60 day to 88 day (*n* = 6)

Item	Control	3 mg/kg DON	6 mg/kg DON	12 mg/kg DON	*P*-value
ADG (g)	421.6 ± 9.84^a^	417.9 ± 51.7^a^	378.8 ± 12.76^a^	236.5 ± 30.5^b^	<0.0001
ADFI (g)	1021.2 ± 17.1^a^	1043.22 ± 104.84^a^	870.1 ± 19^b^	596.1 ± 48.39^c^	<0.0001
F/G (g feed/g gain)	2.43 ± 0.18	2.49 ± 0.32	2.31 ± 0.24	2.52 ± 0.36	0.5964

Results are expressed as means ± SEM for six animals

ADG: average daily weight gain (g/day), ADFI: average daily feed intake (g/day), F/G: feed/gain ratio

^a–c^Values with different letters within the same row are significantly different (*P < 0.05*).

### Relative organ weights

Table 2 shows the effects of dietary DON-contaminated diet on relative organ weights in 60-to 88-day-old pigs. There were no significant differences among the four groups with regard to the spleen, kidney and heart (P>0.05). However, the relative liver weights in the pigs fed the diets with 3 mg/kg DON and 12 mg/kg DON were higher than control. No differences were seen among the DON contaminated diets.

**Table 2:** Relative organ weights (g/kg BW) of growing pigs fed with diets containing deoxynivalenol (DON) contaminated corn (*n* = 6)

Item	Control	3 mg/kg DON	6 mg/kg DON	12 mg/kg DON	*P*-value
Heart	0.53 ± 0.12	0.54 ± 0.14	0.5 ± 0.13	0.57 ± 0.14	0.8366
Liver	2.44 ± 0.13^b^	2.72 ± 0.12^a^	2.7 ± 0.23^a,b^	2.81 ± 0.18^a^	0.0081
Spleen	0.24 ± 0.03	0.2 ± 0.09	0.19 ± 0.02	0.17 ± 0.09	0.3393
Kidney	0.45 ± 0.06	0.5 ± 0.06	0.47 ± 0.04	0.53 ± 0.11	0.2701

Results are expressed as means ± SEM for six animals

^a,b^Values with different letters within the same row are significantly different (*P < 0.05*)

### Serum biochemical parameters and amino acid concentrations

Table 3 shows effects of three dose DON-contaminated diet on serum biochemical parameters of pigs from age 60 to 88 days. There was no difference in the concentrations of albumin (ALB), creatinine (CRE) and serum fasting blood glucose (GLU) between control group and the DON treatment groups (*P*>0.05). The blood urea nitrogen (BUN) values were similar in the 3 mg/kg DON and 6 mg/kg DON groups, but values in the 6 mg/kg DON and 12 mg/kg DON groups were significantly higher than control (*P* < 0.05). The alkaline phosphatase (ALP) activities in 12 mg/kg DON group was significantly higher than in control, 3 mg/kg DON and 6 mg/kg DON groups (*P* < 0.01). The alanine aminotransferase (ALT) activities in 6 mg/kg DON group and 12 mg/kg DON group were significantly higher than control and 3 mg/kg DON group (*P* < 0.01). The aspartate amino transferase (AST) activities in 12 mg/kg DON group was significantly higher than control and 3 mg/kg DON group (*P* < 0.01).

**Table 3:** Serum biochemical chemical parameters of growing pigs fed with diets containing Deoxynivalenol (DON)-contaminated corn (*n* = 6)

Item	Control	3 mg/kg DON	6 mg/kg DON	12 mg/kg DON	*P*-value
ALB (g/L)	35.16 ± 3.46	32.88 ± 5.15	28.95 ± 3.21	26.6 ± 8.75	0.0644
BUN (mmol/L)	5.28 ± 0.51^a^	5.74 ± 0.84^a,b^	6.82 ± 0.72^b,c^	7.1 ± 0.88^c^	0.0011
GLU (mmol/L)	7.93 ± 0.94	8.06 ± 1.03	7.42 ± 0.59	7.58 ± 1.53	0.7135
CRE (mmol/L)	48.23 ± 5.75	52.09 ± 8.24	53.39 ± 8.7	49.35 ± 5.58	0.5878
ALP (U/L)	742.3 ± 34.3^a^	840.91 ± 164.3^a^	938.6 ± 98.3^a^	1236.9 ± 232.2^b^	0.0001
ALT (U/L)	56.67 ± 7.23^a^	54.99 ± 9.25^a^	80.92 ± 7.38^b^	86 ± 11.68^b^	<0.0001
AST (U/L)	93.47 ± 8.94^a^	111.77 ± 18.76^a,b^	133.84 ± 10.63^b,c^	140.34 ± 15.42^c^	<0.0001

Results are expressed as means ± SEM for six animals

ALB: albumin, BUN: blood urea nitrogen, GLU: glucose, CRE: creatinine, ALP: alkaline phosphatase, ALT: alanine aminotransferase, AST: aspartate aminotransferase

^a-c^Values with different letters within the same row are significantly different (*P < 0.05*)

Table 4 shows effects of 3 dose DON-contaminated diet on serum amino acid concentrations of pigs from age 60 to 88 days. The L-arginine concentration in 12 mg/kg DON group was significantly lower than control, 3 mg/kg DON group (*P* < 0.01). The L-histidine concentration in 6 mg/kg DON group and 12 mg/kg DON group were significantly lower than control (*P* < 0.05). The L-lysine concentration in 12 mg/kg DON group was significantly lower than control, 3 mg/kg DON group and 6 mg/kg DON group (*P* < 0.01). The L-threonine concentration in 6 mg/kg DON group and 12 mg/kg DON group were significantly lower than control (*P* < 0.01). The concentrations of L-valine, glycine and L-serine in 12 mg/kg DON group was significantly lower than control and 3 mg/kg DON group (*P* < 0.01). The L-glutamate concentration in 12 mg/kg DON group was significantly lower than control, 3 mg/kg DON and 6 mg/kg DON groups (*P* < 0.01). The L-tyrosine of concentration in 12 mg/kg DON group was significantly lower than control and 3 mg/kg DON groups (*P* < 0.05). The L-aspartate concentration in 12 mg/kg DON group was significantly lower than control and 3 mg/kg DON groups (*P* < 0.05). The L-glutamine concentration in 6 mg/kg group and 12 mg/kg DON group were significantly lower than control (*P* < 0.01). The L-alanine concentration in 12 mg/kg DON group were significantly lower than control and 3 mg/kg DON groups (*P* < 0.01).

**Table 4:** Serum amino acid concentrations of growing pigs fed with diets Deoxynivalenol (DON)-contaminated corn (*n* = 6)

Item	Control	3 mg/kg DON	6 mg/kg DON	12 mg/kg DON	*P*-value
L-arginine	153.36 ± 25.32^a^	162.87 ± 19.74^a^	128.82 ± 25.16^a,b^	100.58 ± 21.64^b^	0.0007
L-histidine	87.26 ± 12.61^a^	74.18 ± 11.53^a,b^	60.17 ± 15.29^b^	62.86 ± 19.25^b^	0.0204
L-isoleucine	122.53 ± 19.25^a^	118.64 ± 15.82^a^	100.73 ± 17.79^a,b^	85.28 ± 11.81^b^	0.0028
L-leucine	183.26 ± 27.16	185.32 ± 20.31	162.58 ± 14.63	162.15 ± 27.38	0.1746
L-lysine	150.03 ± 18.25^a^	138.74 ± 12.69^a^	139.31 ± 23.64^a^	102.98 ± 21.83^b^	0.0028
L-methionine	105.29 ± 18.61	90.78 ± 17.8	93.92 ± 20.51	82.83 ± 28.36	0.3715
L-phenylalanine	89.73 ± 10.12	94.89 ± 23.75	70.93 ± 19.2	71.73 ± 18.28	0.0766
L-threonine	158.07 ± 29.71^a^	128.73 ± 11.83^a,b^	118.39 ± 23.7^b^	105.72 ± 18.32^b^	0.0037
L-tryptophan	128.96 ± 23.98	137.26 ± 12.82	121.92 ± 10.26	117.29 ± 9.96	0.1575
L-valine	169.38 ± 10.71^a^	152.77 ± 16.89^a,b^	129.19 ± 17.25^b,c^	110.62 ± 21.25^c^	<0.0001
Glycine	279.49 ± 20.19^a^	205.01 ± 24.75^b^	172.96 ± 26.61^b,c^	152.84 ± 16.97^c^	<0.0001
L-serine	196.18 ± 24.13^a,b^	221.03 ± 17.11^a^	162.2 ± 10.29^b,c^	151.65 ± 29.57^c^	<0.0001
L-glutamate	260.25 ± 20.46^a^	232.82 ± 18.52^a,b^	218.71 ± 22.39^b^	131.39 ± 32.05^c^	<0.0001
L-tyrosine	102.93 ± 15.39^a,b^	112.25 ± 8.28^a^	105.19 ± 18.27^a,b^	81.59 ± 21.37^b^	0.0258
L-asparagine	72.21 ± 9.65	79.23 ± 19.07	59.74 ± 7.57	60.93 ± 11.94	0.0458
L-aspartate	68.2 ± 12.38^a^	65.84 ± 12.53^a^	54.75 ± 15.56^a,b^	41.99 ± 14.7^b^	0.0144
L-glutamine	351.92 ± 27.78^a^	302.58 ± 29.43^a,b^	274.82 ± 31.84^b^	251.02 ± 42.75^b^	0.0003
L-cysteine (free)	119.27 ± 13.29	131.59 ± 25.98	120.84 ± 23.17	106.35 ± 17.01	0.2374
L-alanine	392.96 ± 41.12^a^	355.29 ± 46.08^a^	317.84 ± 65.58^a,b^	270.12 ± 50.95^b^	0.0038
L-proline	86.25 ± 15.79	70.86 ± 21.52	68.63 ± 9.82	59.9 ± 22.76	0.1227

Values are μmol/l. Serum amino acid levels were determined by HPLC Ultimate 3000 and 3200 Q TRAP LC–MS/MS. Results are expressed as means ± SEM for six animals

^a-c^Values with different letters within the same row are significantly different (*P < 0.05*)

### Serum hormonal components

Table 5 shows dietary effects on serum growth hormone (GH), insulin-like growth factor 1 (IGF1), total superoxide dismutase (T-SOD), haptoglobin (HP) and glutathione peroxidase (GSH-Px) concentrations. The serum activity of GH in 12 mg/kg DON group was significantly lower than 3 mg/kg DON group (*P* < 0.05). The serum activity of IGF1 in control group and 3 mg/kg DON group were significantly lower than those 2 groups (*P* < 0.01). The serum activity of T-SOD in 12 mg/kg DON group were significantly lower than control and 3 mg/kg DON group (*P* < 0.01). The HP in control group and 3 mg/kg DON group were significantly lower than 6 mg/kg DON group and 12 mg/kg DON group (*P* < 0.01). The GSH-Px in 6 mg/kg DON group and 12 mg/kg DON group were significantly lower than control and 3 mg/kg DON group (*P* < 0.01).

**Table 5:** Serum hormonal characters of growing pigs fed with diets containing Deoxynivalenol (DON)-contaminated corn (*n* = 6)

Item	Control	3 mg/kg DON	6 mg/kg DON	12 mg/kg DON	*P*-value
GH (ng/ml)	29.01 ± 0.88^a,b^	29.86 ± 4.13^a^	25.99 ± 0.64^a,b^	24.03 ± 4.55^b^	0.0145
IGF1 (pg/ml)	92.18 ± 2.51^a^	90.45 ± 5.02^a^	81.03 ± 2.62^b^	80.47 ± 6.47^b^	0.0001
T-SOD (U/ml)	117.5 ± 6.26^a^	100.88 ± 14.24^a,b^	84.48 ± 8.97^b,c^	79.94 ± 15.62^c^	<0.0001
HP (μg/ml)	8.87 ± 1.95^a^	10.26 ± 1.39^a^	13.42 ± 1.02^b^	13.71 ± 1.04^b^	<0.0001
GSH-Px (U/ml)	3424.7 ± 147.5^a^	3388.38 ± 294.5^a^	2967.3 ± 124.4^b^	2521.3 ± 334.6^c^	<0.0001

Results are expressed as means ± SEM for six animals

GH: growth hormone, IGF1: insulin-like growth factor 1, T-SOD: total superoxide dismutase, HP: haptoglobin, GSH-Px: glutathione peroxidase

^a-c^Values with different letters within the same row are significantly different (*P < 0.05*)

### Jejunal morphology

**Fig. 1:**
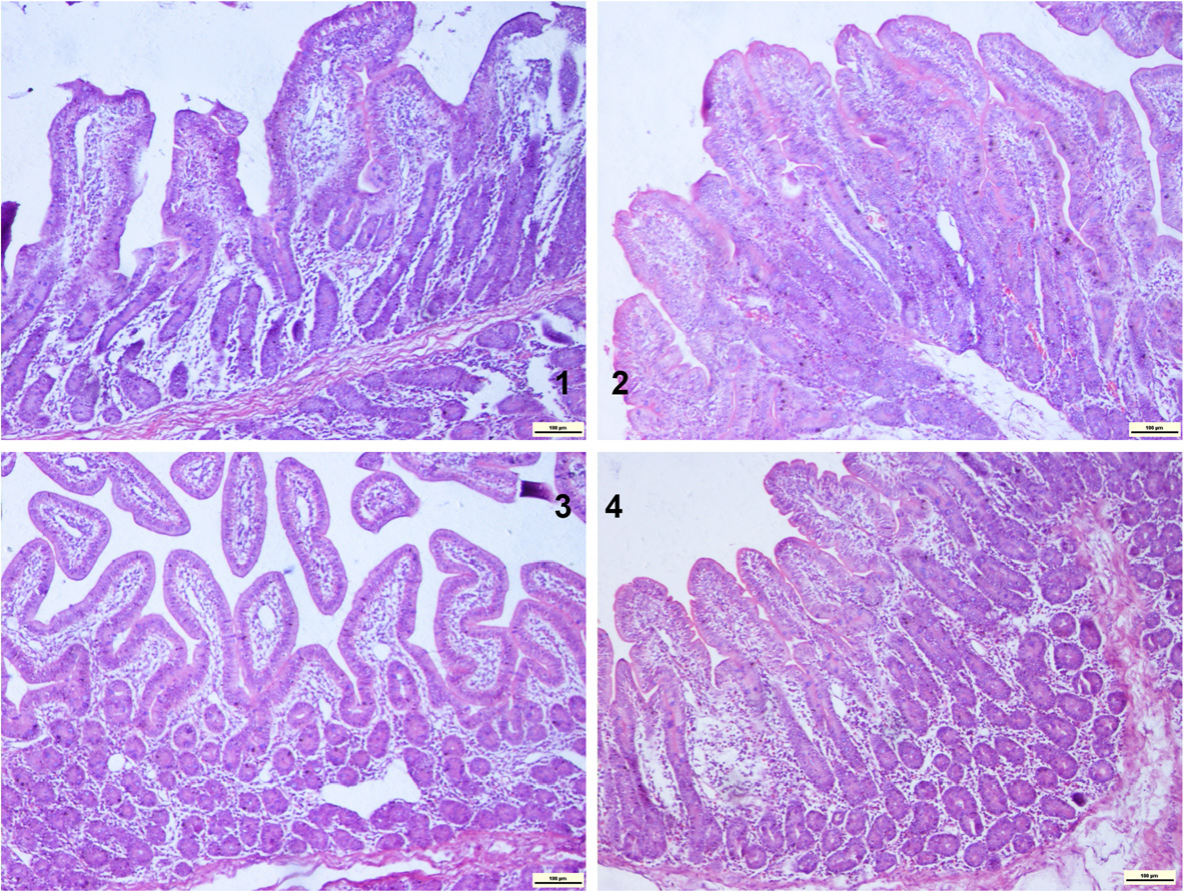
The Jejunal morphology (HE × 100) changes of in growing pigs fed with three different dose Deoxynivalenol (DON) contaminated diet. Pigs in the control group (**Panel 1**) and 6 mg/kg DON group (**Panel 3**) were fed a DON uncontaminated diet and a 6 mg/kg DON contaminated diet, respectively. Pigs in the 3 mg/kg DON (**Panel 2**) and 12 mg/kg DON group (**Panel 4**) were fed a 3 mg/kg DON contaminated diet and a 12 mg/kg DON contaminated diet, respectively. The scale bars in Fig. 1 represent 100 μm

No abnormal morphology was observed for the jejunal morphology in the control (Fig. 1). Table 6 shows the jejunal morphology changes of growing pigs fed with 3 dose DON-contaminated diet. The villus height of the jejunal in control and 3 mg/kg DON group were significantly higher than 6 mg/kg DON group and 12 mg/kg DON group (*P* < 0.05). The crypt depth of the jejunal was no significant differences among the 4 groups. The lymphocyte number of the jejunal in control was significantly lower than 12 mg/kg DON group (*P* < 0.05).

**Table 6:** The Jejunal morphology changes of growing pigs fed with diets Deoxynivalenol (DON)-contaminated corn (*n* = 6)

Item	Control	3 mg/kg DON	6 mg/kg DON	12 mg/kg DON	*P*-value
Villus height (μM)	311.7 ± 26.9^a^	296.3 ± 33.1^a^	290.5 ± 26.2^a,b^	259.9 ± 19.8^b^	0.024
Crypt depth (μM)	119.9 ± 16.4	130.2 ± 23.8	142.6 ± 28.3	148.6 ± 26	0.2
Goblet cell number	15.17 ± 2.93^a^	12.67 ± 2.88^a,b^	10.5 ± 2.74^b^	12.17 ± 4.54^a,b^	0.147
Lymphocyte number	115 ± 34.3 ^b^	131.7 ± 35.5^a,b^	152.2 ± 34.5^a,b^	173.3 ± 26.9^a^	0.034

Results are expressed as means ± SEM for six animals

^a,b^Values with different letters within the same row are significantly different (*P < 0.05*)

### mRNA expression of nutrients transporter genes

Eight critical intestinal amino acid transporters (excitatory amino acid transporter-3 (EAAT-3), b^0,+^amino acid transporter (B^0,+^AT), sodium-glucose transporter-1 (SGLT-1), glucose transporter-2 (GLUT-2), dipeptide transporter-1 (PepT-1), Na^+^-dependent neutral amino acid exchanger-2 (ASCT-2), cationic amino acid transporter-1 (CAT-1) and y^+^L-type amino acid transporter-1 (LAT-1)) mRNA expressions were tested at the end of feeding, and the results were shown in the Table 7. The mRNA expressions level of the EAAT3 in control was significantly higher than in the 3 DON treatment groups (*P* < 0.01). The mRNA expression levels of the B^0,+^AT, GLUT-2 and ASCT-2 were no significantly different among the 4 groups. The mRNA expressions level of the SGLT-1 in control and 3 mg/kg DON group were significantly higher than 12 mg/kg DON group (*P* < 0.01). The mRNA expressions level of the PepT-1 in 3 mg/kg DON group was significantly higher than 6 mg/kg DON group and 12 mg/kg DON group (*P* < 0.01). The mRNA expressions level of the CAT-1 and LAT-1 in control and 3 mg/kg DON group were significantly higher than 6 mg/kg DON group and 12 mg/kg DON group (*P* < 0.01).

**Table 7:** The intestinal nutrients transporter genes of growing pigs fed with Deoxynivalenol (DON)-contaminated corn (*n* = 6)

Item	Control	3 mg/kg DON	6 mg/kg DON	12 mg/kg DON	*P*-value
EAAT-3	1 ± 0.06^a^	0.84 ± 0.11^a,b^	0.69 ± 0.17^b^	0.61 ± 0.21^b^	0.0011
B^0,+^AT	1 ± 0.15	0.92 ± 0.21	0.73 ± 0.26	0.75 ± 0.21	0.1057
SGLT-1	1 ± 0.08^a,b^	1.02 ± 0.13^a^	0.76 ± 0.15^b,c^	0.72 ± 0.23^c^	0.0044
GLUT-2	1 ± 0.08	0.86 ± 0.15	0.78 ± 0.05	0.81 ± 0.25	0.0956
PepT-1	1 ± 0.12^a,b^	1.09 ± 0.13^a^	0.87 ± 0.09^b^	0.79 ± 0.16^b^	0.0028
ASCT-2	1 ± 0.19	0.94 ± 0.24	0.98 ± 0.07	0.82 ± 0.2	0.3625
CAT-1	1 ± 0.15^a^	0.81 ± 0.15^a^	0.48 ± 0.06^b^	0.5 ± 0.18^b^	<0.0001
LAT-1	1 ± 0.07^a^	0.89 ± 0.19^a^	0.41 ± 0.06^b^	0.46 ± 0.09^b^	<0.0001

Results are expressed as means ± SEM for six animals

EAAT-3: excitatory amino acid transporter-3, B^0,+^AT: b^0,+^amino acid transporter, SGLT-1: sodium-glucose transporter-1, GLUT-2: glucose transporter-2, PepT-1: dipeptide transporter-1, ASCT-2: Na^+^-dependent neutral amino acid exchanger-2, CAT-1: cationic amino acid transporter-1, LAT-1: y^+^L-type amino acid transporter-1

^a–c^Values with different letters within the same row are significantly different (*P < 0.05*)

## Discussion

DON is a common contaminant of cereal crops like wheat, barley, corn and oats and of high importance in food industry, and increasingly a food safety issue problem worldwide. Understanding of variable DON toxicology also requires the systematic combination of growth performance, serum biochemical profile, jejunal morphology, especially for the expression different nutrient transporter genes when pigs are exposed to different doses of DON and coherent responses on animal or human health. Considering that the gastrointestinal tract and the immune system of pigs are not vastly different that of humans, the pig can be regarded as a good model that can be applied to humans [25]. In the present study when pigs were fed 3 dose DON-containing diets, especially significantly in 12 mg/kg DON group compared with other 3 groups (*P* < 0.01). Consistent with previous studies examining the impact of dietary DON, feed intake was significantly reduced in a dose-dependent manner (Table 1) [18, 26–34]. This finding supports the hypothesis that the adverse effects of DON contaminated diets on growth performance of growing pigs is primarily caused by depressing the voluntary feed intake [34–40].

In the present study, the relative liver weight in the 12 mg/kg DON treatment group was significantly greater than that in the control (*P*<0.01), but there were no significant differences in other organs (Table 2). The report demonstrated that there were no changes in organ weights with the use of DON concentrations ranging from 750 to 3000 μg/kg [39]. It is not consistent with Chaytor and co-workers, which analyses the effect of the combination of DON and aflatoxins on the weights of internal organs, and found no changes in the weights of the liver, kidney or spleen [7]. The organ weights related to live weight seem to be more appropriate for interpreting the DON effects. A possible explanation for discrepancies between this latter study and our present study could be that the effect of DON on relative organ weights are dependent on the age of pigs, duration of exposure of pigs to DON and the dose of DON [35].

The serum levels of ALB, BUN, GLU, CRE, ALP, ALT and AST were tested as a reflection of the metabolism and visceral organ status of pigs (Table 3). There was no difference in ALB levels, CRE level and GLU level between control and 3 DON treatment groups (P>0.05). It is consistent with previous result showing that dietary exposure to DON has no significant effect on plasma protein concentrations (total protein, albumin and fibrinogen) [41], however, and it is not consistent with previous results showing that the decreased albumin levels have been found in pigs fed a DON-contaminated diet [36]. The increase in ALP in toxin control reflected the abnormal excretion of liver metabolites due to DON-induced systemic toxicity, as described in a previous study [7]. Serum AST and ALT levels have been reported to be sensitive indicators of liver injury, since an increase in these values reflects leakage from injured hepatocytes [42]. The increase in AST and ALT in the 3 DON treatment groups compared with control indicated that this injured mechanism is triggered after DON diet intake, and the result was consistent with relative live weights (Table 2).

Amino acids play important roles as metabolic intermediates in nutrition, immune response, and growth performance [43]. In the present work, we observed that concentrations of L-valine, glycine, L-serine, and L-glutamine in serum were significant decreased by the exposure to DON in feedstuffs, especially significant in 12 mg/kg DON group (*P*<0.01). It is not consistent with our previously reports showing that the dietary supplementation with functional nutrients in a single dose DON exposure [17–19]. A possible reason was the degradation of dietary valine, glycine, glutamine, and serine by the small intestine is increased by 3 dose DON-contaminated food, resulting in their deficiencies in the animals. Increasing evidence shows that these amino acids are very important for tissue protein synthesis and metabolic regulation [43–45].

Antioxidant enzymes comprise a major defense system for preventing organ injury due to excessive quantities of reactive oxygen species that attack proteins, lipids, and DNA, such as T-SOD, HP and GSH-Px [46, 47]. Most of the studies have demonstrated that some mycotoxins can contribute to oxidative stress in cells [48–50]. In the present study, we found that the activity of T-SOD, GSH-Px in the pig serum was markedly decreased after exposure to DON contaminated diets, indicating that DON resulted in oxidative stress in the whole body (Table 5). For the changes of GH and IGF1 in our study, only IGF1 levels were markedly decreased after exposure to DON contaminated diets (Table 5), which is consistent with previously reports [51, 52]. A possible mechanism for the DON-induced IGF-1 suppression involves the induction of IL-6 and other proinflammatory cytokines, which down-regulates the sensitivity of the GH receptor via suppressor of cytokines (SOCS)-and signal transducers and activator of transcription (STAT)-related mechanisms [53, 54].

The main morphological and histological effects observed included villi flattening and shortening, apical necrosis hyperemia and a reduction in the number of goblet cells and lymphocytes (Fig. 1 and Table 6). The villi height reduction indicate that DON resulted in malabsorption and impairment in the jejunum. Similar changes were observed during in vivo and ex vivo exposure of the intestine to DON [55, 56]. The toxic effects of DON are mediated via the inhibition of protein synthesis, thus primarily affecting rapidly dividing cells such epithelial and immune cells [1, 2]. Thus, the observed villi flattening and shortening in the jejunum is probably due to the impairment of cell proliferation as as shown in the Figs. 1. A hyperplasia of intestinal goblet cells has been observed in piglets and broiler chicks receiving feed contaminated with 30 and 300 mg FB_1_/kg feed, respectively [57, 58]. In the present study, a slightly decrease in the number of goblet cells and a increase lymphocyte cells were observed (Table 6). Intestinal mucus protects the epithelium against adhesion and invasion by pathogens, therefore, a increment in the number of lymphocyte cells can affect the intestinal barrier function [18, 19, 59, 60].

The absorption of amino acids mainly depends on their transporters on the membrane of the enterocyte [17, 61]. The mRNA expressions level of the B^0,+^AT, GLUT-2 and ASCT-2 were no significant differences among the 4 groups, intestinal protein levels for these transporters need to be quantified using western blot techniques [62]. The EAAC-3 is a sodium dependent glutamate transporter that can transport neutral amino acids, especially glutamate and cysteine, into intestinal vesicles [63]. The SGLT-1, which appears gradually on the apical membrane during the differentiation of enterocytes, but in mature enterocytes, the SGLT-1 is the main sugar transport system [64, 65]. In the piglet small intestine, PepT-1 mainly transports dipeptides and tripeptides from the digestion of dietary proteins [66]. In addition, CAT-1 transports cationic amino acids in the kidney and the small intestine, whereas LAT-1 transporter is a mediator of cationic amino acid efflux from epithelial cells [67]. In the present study, the mRNA expressions level of EAAC-3, SGLT-1, PepT-1, CAT-1 and LAT-1 in control were slightly or significantly higher than in the in the 3 DON-containing treatment groups. The SGLT-1 data, unlike that of GLUT-2, in the present study was consistent with that of study of Maresca et al. [11]. The EAAC and CAT-1 data were not consistent with our previous data obtained with a single dose of DON exposure [18]. Combined with the results of the present study and previously published data, it is clear that DON can decrease absorption of glucose, amino acid and peptide by inhibiting the mRNA expression of the relevant transporters [17–19]. However, the mRNA expression of amino acid transporters in the amino acid absorption ratio did not strictly correspond to the changes in amino acid intake [18]. This relationship between amino acid expression and amino acid intake in DON-contaminated pigs will require further investigation.

## Conclusions

In conclusion, feeding DON-contaminated diets to growing pigs significantly reduced feed intake resulting in decreased growth performance. This further altered serum biochemical and amino acid profiles, jejunal morphology, and the mRNA expression of nutrients transporter genes. Therefore, dietary DON can selectively decreased the mRNA expression of nutrient transporter genes in growing pigs.

## Methods

### Ethics statement

This study was conducted according to the guidelines of the Declaration of Helsinki and all procedures involving animal subjects were approved by the animal welfare committee of the Institute of Subtropical Agriculture, Chinese Academy of Sciences (Changsha, Hunan Province, China).

### Preparation of mouldy corn

*Fusarium graminearum* isolate R6576 was obtained from the College of Plant Science & Technology of Huazhong Agricultural University (Wuhan, Hubei Province, China). Preparation, cultivation and collection of fungus from mouldy corn was performed as described previously [17–19]. In brief, water was added to a non-contaminated basal diet until it reached 20 % moisture. The wet feed was then cultured under ambient conditions (temperature 23–28 °C, humidity 68–85 %) until mildew was clearly observed. Finally, the mold contaminated diet was naturally air-dried, mixed, and sampled for detection of mycotoxins. The contents of mycotoxins in mould-contaminated feed were detected by liquid chromatography as described previously (Beijing Taileqi, Beijing, China) (Table 8) [17–19].

**Table 8:** Mycotoxin content in contaminated and non-contaminated feed mixture

Catalogue	AFB_1_ (ppb)	ZEN (ppm)	OCH (ppb)	FB_1_ (ppm)	T-2 (ppm)	DON (ppm)
Limit of detection	0.05	0.01	0.5	0.05	0.1	0.1
Basal feed	undetected	0.863	3.74	0.65	undetected	0.52
Contaminated feed	undetected	0.697	4.63	0.74	undetected	

The contents of mycotoxins in the diet were detected by chromatograph of liquid (Beijing Taileqi, Beijing, China)

AFB_1_: aflatoxin B_1_; ZEN: zearalenone; OCH: ochratoxins; DON: deoxynivalenol; FB_1_: fumonisins B_1_

### Pigs management and sample collection

A total of twenty-four 60 day-old healthy growing pigs (Landrace × Large × Yorkshire) (Zhenghong Co., Ltd., Hunan Province, China) with a mean body weight of 16.3 ± 1.5 kg were randomly assigned to 4 dietary treatments: (1) a DON-free diet (control); (2) a diet with 3 mg DON/kg; (3) a diet with 6 mg DON/kg diet; and (4) a diet with 12 mg DON/kg of diet. There were 6 pigs per group (three male; three female). All diets were formulated to meet the National Research Council (1998) recommended nutrient requirements for growing pigs. The ingredient and nutrient composition of the diets is as reported by our previously report [18]. Before the pigs were challenged with DON, pigs were allowed to acclimatize to the housing conditions with access to a commercial diet with 1.64 % Alanine as isonitrogenous control for 7 days. Pigs had free access to drinking water and their respective diets throughout the experimental period. After 21 days of dietary exposure to DON, and immediately after electrical stunning, the pigs were killed for analysis. Body weight and feed consumption were recorded.

After 21 days of dietary exposure to DON, 5 mL of blood was collected aseptically in tubes from a jugular vein 2 h after feeding, centrifuged at 3000 × g for 10 min at 4 °C to obtain serum samples, and stored at –80 °C for further analysis. The liver, spleen, kidney and heart were removed and weighed. The weights were recorded both as the organ weight and the weight as a percent of the total body weight. The small intestine was rinsed thoroughly with ice-cold physiological saline solution (PBS) and the jejunum and ileum were dissected.

### Analysis of serum biochemical parameters and amino acid profile

Serum biochemical parameters, including GLU, ALB, ALT, AST, BUN, CRE, and ALP, were measured using spectrophotometric kits in accordance with the manufacturer’s instructions (Nanjing Jiangcheng Biotechnology Institute, Jiangsu Province, China) and determined using an Automatic Biochemistry Radiometer (Au640, Olympus).

Twenty amino acids in serum were determined by LC–MS/MS (HPLC Ultimate3000 and 3200 QTRAP LC–MS/MS) as described previously [68].

### Analysis of serum hormonal components

GH, IGF1, T-SOD, HP and GSH-Px were measured with the use of ELISA test kits (Beijing Laboratory Biotech Co., LTD, China).

### Determination of jejunal morphology

Segments (2 cm) of the jejunum were cut and fixed in 4 % neutral buffered 10 % formalin, processed using routine histological methods, and mounted in paraffin blocks [17, 69]. Six-micrometer-thick sections were cut and stained with hematoxylin and eosin (H&E). After dehydration, embedding, sectioning, and staining, villous height, crypt depth, and goblet cell and lymphocyte counts were measured with computer-assisted microscopy (Micrometrics TM; Nikon ECLIPSE E200, Tokyo, Japan).

### Quantification of nutrients transporter genes mRNA

Total RNA was isolated from liquid nitrogen-pulverized intestine tissue sample with TRIzol regent (Invitrogen, Carlsbad, CA, USA) and then treated with DNase I (Invitrogen) according to the manufacturer’s instructions. The quality of RNA was checked by 1 % agarose gel electrophoresis after staining with 10 μg/ml ethidium bromide. The RNA had an OD_260_:OD_280_ ratio between 1.8 and 2.0. First-strand cDNA was synthesized with oligo (dT) 20 and Superscript II reverse transcriptase (Invitrogen, USA).

Primers were designed with Primer 5.0 based on the cDNA sequence of the pig to produce an amplification product (Table 9). β-actin was used as a housekeeping gene to normalize target gene transcript levels. Real-time PCR analysis was performed as described previously [18]. In brief, 2 μL of cDNA template was added to a total volume of 25 μL containing 12.5 μL SYBR Green mix and 1 μmol/l each of forward and reverse primers. We used the following protocol: (i) pre-denaturation (10 s at 95 °C); (ii) amplification and quantification, repeated 40 cycles (5 s at 95 °C, 20 s at 60 °C); (iii) melting curve (60–99 °C with a heating rate of 0.1 °C S-1 and fluorescence measurement). The relative levels of genes were expressed as a ratio of mRNA as R = 2^-(∆∆Ct)^. The efficiency of real-time PCR was determined by the amplification of a dilution series of cDNA according to the equation 10^(-1/slope)^, and the results for target mRNA were consistent with those for β-actin. Negative controls were created by replacing cDNA with water.

**Table 9:** Primers used for RT-PCR

Target gene	Primer sequence	Accession NO.	Size (bp)
B^0,+^AT	Sense 5′-GCGAGTACCCGTACCTGATG-3′	NM_001110171.1	173
B^0,+^AT	Antisense 5′-TTTCACGACGACTTGAGGGG-3′		
SGLT-1	Sense 5′-TCATCATCGTCCTGGTCGTCTC-3′	M34044.1	144
SGLT-1	Antisense 5′-CTTCTGGGGCTTCTTGAATGTC-3′		
GLUT-2	Sense 5′-ATTGTCACAGGCATTCTTGTTAGTCA-3′	NM_001097417	273
GLUT-2	Antisense 5′-TTCACTTGATGCTTCTTCCCTTTC-3′		
y^+^LAT-1	Sense 5′-TTCTCTTACTCGGGCTGGGA-3′	EU047705.1	400
y^+^LAT-1	Antisense 5′-GCGCCATGAGACCATTGAAC-3′		
CAT-1	Sense 5′-GCTGTCATGGCCTTCCTCTT-3′	NM_001012613.1	138
CAT-1	Antisense 5′-CTGGTACACCATGTTCGGCT-3′		
GADPH	Sense 5′-AAGGAGTAAGAGCCCCTGGA-3′	DQ845173	140
GADPH	Antisense 5′-TCTGGGATGGAAACTGGAA-3′		
EAAT-3	Sense 5′-TTGGGCATTGGGCAGATCAT-3′	JF521497.1	187
EAAT-3	Antisense 5′-TCACCATGGTCCTGAAACGG-3′		
ASCT-2	Sense 5′-CTGGTCTCCTGGATCATGTGG-3′	DQ231578.1	172
ASCT-2	Antisense 5′-CAGGAAGCGGTAGGGGTTTT-3′		
PepT-1	Sense 5′-CAGACTTCGACCACAACGGA-3′	NM_214347.1	99
PepT-1	Antisense 5′-TTATCCCGCCAGTACCCAGA-3′		

EAAT-3: excitatory amino acid transporter-3, B^0,+^AT: b^0,+^amino acid transporter, SGLT-1: sodium-glucose transporter-1, GLUT-2: glucose transporter-2, PepT-1: dipeptide transporter-1, ASCT-2: Na^+^-dependent neutral amino acid exchanger-2, CAT-1: cationic amino acid transporter-1, LAT-1: y^+^L-type amino acid transporter-1

### Statistical analysis

Statistical analyses were performed with the SPSS17.0 software (Chicago, IL, USA) [17, 19]. Data were subjected to one-way analysis of variance followed by the Duncan’s multiple comparisons test. Values are expressed as the mean ± standard error of the mean (SEM).

## Abbreviations

DON: Deoxynivalenol
ADG: Average daily gain
ADFI: Average daily feed intake
F/G: Feed/gain ratio
GIT: Gastro-intestinal tract
PBS: Physiological saline solution
GLU: Serum fasting blood glucose
ALB: Albumin
ALT: Alanine aminotransferase
AST: Aspartate amino transferase
BUN: Blood urea nitrogen
CRE: Creatinine
ALP: Alkaline phosphatase
GH: Growth hormone
IGF1: Insulin-like growth factor 1
T-SOD: Total superoxide dismutase
HP: Haptoglobin
GSH-Px: Glutathione peroxidase
H&E: Hematoxylin and eosin
EAAT-3: Excitatory amino acid transporter-3
B^0,+^AT: B^0,+^amino acid transporter
SGLT-1: Sodium-glucose transporter-1
GLUT-2: Glucose transporter-2
PepT-1: Dipeptide transporter-1
ASCT2: Na^+^-dependent neutral amino acid exchanger-2
CAT-1: Cationic amino acid transporter-1
LAT-1: y^+^ L-type amino acid transporter-1
AFB_1_: Aflatoxin B_1_
ZEN: Zearalenone
OCH: Ochratoxins
FB_1_: Fumonisins B_1_
